# The differential activation of cardiovascular hormones across distinct stages of portal hypertension predicts clinical outcomes

**DOI:** 10.1007/s12072-021-10203-9

**Published:** 2021-05-21

**Authors:** Lukas Hartl, Mathias Jachs, Christopher Desbalmes, Dunja Schaufler, Benedikt Simbrunner, Rafael Paternostro, Philipp Schwabl, David Josef Maria Bauer, Georg Semmler, Bernhard Scheiner, Theresa Bucsics, Ernst Eigenbauer, Rodrig Marculescu, Thomas Szekeres, Markus Peck-Radosavljevic, Stefan Kastl, Michael Trauner, Mattias Mandorfer, Thomas Reiberger

**Affiliations:** 1 Division of Gastroenterology and Hepatology, Department of Medicine III, Medical University of Vienna, Waehringer Guertel 18-20, 1090 Vienna, Austria; 2 Vienna Hepatic Hemodynamic Lab, Medical University of Vienna, Vienna, Austria; 3 Christian Doppler Lab for Portal Hypertension and Liver Fibrosis, Medical University of Vienna, Vienna, Austria; 4 IT-Systems and Communications, Medical University of Vienna, Vienna, Austria; 5 Department of Laboratory Medicine, Medical University of Vienna, Vienna, Austria; 6 Department of Internal Medicine and Gastroenterology (IMuG), Hepatology, Endocrinology, Rheumatology and Nephrology, Central Emergency Medicine (ZAE), Klinikum Klagenfurt am Wörthersee, Klagenfurt, Austria; 7 Division of Cardiology, Department of Medicine II, Medical University of Vienna, Vienna, Austria

**Keywords:** Renin, Natriuretic peptide, brain, Arginine vasopressin, proBNP, Copeptin, Renin/angiotensin/aldosterone system, Hyperdynamic circulation, Cirrhosis, Ascites, Decompensation

## Abstract

**Background and aims:**

The cardiovascular hormones renin/angiotensin/aldosterone (RAA), brain-type natriuretic peptide (BNP)and arginine-vasopressin (AVP) are key regulators of systemic circulatory homeostasis in portal hypertension (PH). We assessed (i) the activation of renin, BNP and AVP across distinct stages of PH and (ii) whether activation of these hormones correlates with clinical outcomes.

**Methods:**

Plasma levels of renin, proBNP and copeptin (AVP biomarker) were determined in 663 patients with advanced chronic liver disease (ACLD) undergoing hepatic venous pressure gradient (HVPG) measurement at the Vienna General Hospital between 11/2011 and 02/2019. We stratified for Child stage (A–C), HVPG (6–9 mmHg, 10–15 mmHg, ≥ 16 mmHg) and compensated vs. decompensated ACLD.

**Results:**

With increasing PH, hyperdynamic state was indicated by higher heart rates (6–9 mmHg: median 71.0 [IQR 18.0] bpm, 10–15 mmHg: 76.0 [19.0] bpm, ≥ 16 mmHg: 80.0 [22.0] bpm; *p* < 0.001), lower mean arterial pressure (6–9 mmHg: 103.0 [13.5] mmHg, 10–15 mmHg: 101.0 [19.5] mmHg, ≥ 16 mmHg: 99.0 [21.0] mmHg; *p* = 0.032) and lower serum sodium (6–9 mmHg: 139.0 [3.0] mmol/L, 10–15 mmHg: 138.0 [4.0] mmol/L, ≥ 16 mmHg: 138.0 [5.0] mmol/L; *p* < 0.001). Across HVPG strata (6–9 mmHg vs. 10–15 mmHg vs ≥ 16 mmHg), median plasma levels of renin (21.0 [50.5] vs. 25.1 [70.9] vs. 65.4 [219.6] µIU/mL; *p* < 0.001), proBNP (86.1 [134.0] vs. 63.6 [118.0], vs. 132.2 [208.9] pg/mL; *p* = 0.002) and copeptin (7.8 [7.7] vs. 5.6 [8.0] vs. 10.7 [18.6] pmol/L; *p* = 0.024) increased with severity of PH. Elevated renin levels independently predicted first hepatic decompensation (adjusted hazard ratio [aHR]: 1.69; 95% confidence interval [95% CI] 1.07–2.68; *p* = 0.025) and mortality in compensated patients (aHR: 3.15; 95% CI 1.70–5.84; *p* < 0.001) and the overall cohort aHR: 1.42; 95% CI 1.01–2.01; *p* = 0.046). Elevated copeptin levels predicted mortality in decompensated patients (aHR: 5.77; 95% CI 1.27–26.33; *p* = 0.024) and in the overall cohort (aHR: 3.29; 95% CI 1.36–7.95; *p* = 0.008). ProBNP levels did not predict clinical outcomes.

**Conclusions:**

The cardiovascular hormones renin, proBNP and AVP are activated with progression of ACLD and PH. Renin activation is a risk factor for hepatic decompensation and mortality, especially in compensated patients. Increased plasma copeptin is a risk factor for mortality, in particular in decompensated patients.

**Supplementary Information:**

The online version contains supplementary material available at 10.1007/s12072-021-10203-9.

## Introduction

Advanced chronic liver disease (ACLD) causes considerable morbidity and mortality word-wide [1]. Portal hypertension (PH) drives the development of hepatic decompensation and thus plays a pivotal role in ACLD progression [2–4]. Clinically, PH is defined as hepatic venous pressure gradient (HVPG) ≥ 6 mmHg, although PH-related complications occur mostly in patients with HVPG ≥ 10 mmHg, which denotes clinically significant PH (CSPH) [5]. Furthermore, HVPG ≥ 16 mmHg is linked to increased risk of hepatic decompensation, as well as mortality in decompensated cirrhosis [6].

PH in ACLD is caused by both increased intrahepatic vascular resistance and hyperdynamic circulation. An abundance of vasodilators results in increased inflow into the portal venous system, aggravating PH [7]. At the same time, a decrease in peripheral vascular resistance is compensated by elevated cardiac output to maintain mean arterial pressure (MAP), which also promotes splanchnic hyperemia [8].

The renin/angiotensin/aldosterone (RAA) system, brain-type natriuretic peptide (BNP) and arginine-vasopressin (AVP) not only represent key regulators of circulatory homeostasis, but also play an important role in PH and hyperdynamic circulation [9–11]. While AVP production is primarily regulated by central osmoreceptors sensing plasma osmolality [12], BNP is released by stretching of the myocardium [13]. The regulation of renin secretion is complex, as it is triggered by a decrease in MAP (baroreceptor stimulation), a decrease of sodium concentration in the distal tubule (macula densa stimulation), or sympathetic nervous system activity (beta-1-adrenergic stimulation) [14]. In 1980, Bosch et al. showed that plasma renin activity directly correlates with wedged hepatic venous pressure (WHVP) [15], suggesting a link between RAA activation and PH, and there is overwhelming evidence of increased levels of renin in cirrhotic patients with ascites [15–17]. Furthermore, previous studies have indicated that increased levels of renin and copeptin, an AVP biomarker, may indicate increased risk for mortality in ACLD patients [17–20]. However, these three systems of circulatory homeostasis are yet to be systematically investigated considering both the severity of liver dysfunction and of PH.

The objectives of this study were to assess the activation of renin, proBNP and AVP in distinct stages of (i) liver dysfunction, i.e. MELD/Child strata, (ii) of portal hypertension, i.e. HVPG strata, and (iii) to investigate whether alterations of these systems correlate with the risk for clinical events.

## Methods

### Study population

Patients with ACLD undergoing measurement of HVPG at the Vienna General Hospital between 11/2011 and 02/2019 showing PH were included. At the time of HVPG measurement, hemodynamic parameters (heart rate, MAP) were recorded and blood samples were withdrawn in supine position after the patients rested for at least 30 min. Notably, as HVPG measurement is usually performed as an outpatient procedure, most of our patient were not hospitalized at the time of catheterization.

Patients on non-selective beta-blockers, with invalid HVPG measurements and lack of critical clinical or laboratory data were excluded. Patients after liver transplantation (LT) or transjugular intrahepatic portosystemic shunt (TIPS), with cardiac cirrhosis or hepatocellular carcinoma (HCC) out of Milan criteria were excluded.

Patients were stratified for CTP stage, for MELD 6–9, 10–15 and ≥ 16 points, and for HVPG (6–9 mmHg, 10–15 mmHg, ≥ 16 mmHg). Etiology of ACLD, comorbidities (arterial hypertension, diabetes mellitus, coronary heart disease, heart failure), age, presence of varices, HCC and concomitant medication were evaluated by chart review. Furthermore, hepatic decompensation events including (i) variceal bleeding, (ii) admission due to overt hepatic encephalopathy (HE) and (iii) development or worsening of ascites were recorded. The date of LT, death, and last follow-up was documented.

Further information on measurement of HVPG, laboratory parameters and statistical analysis is provided in the supplementary material.

### Ethics

The study was approved by the ethics committee (EC) of the Medical University of Vienna (1493/2016) and performed according to the current version of the Helsinki Declaration. Due to the retrospective design of the study the EC waived the need for informed consent.

## Results

### ACLD study population (Table 1)

**Table 1:** Patient characteristics and comparison between HVPG strata

Patient characteristics	All patients (*n* = 663)	HVPG			*p* value
		6–9 mmHg (*n* = 114)	10–15 mmHg (*n* = 170)	≥ 16 mmHg (*n* = 379)	
Sex, male/female (% male)	452/211 (68.2%)	83/31 (72.8%)	111/59 (65.3%)	258/121 (68.1%)	0.411
Age, years (IQR)	56.6 (15.5)	55.0 (15.5)	56.7 (14.5)	57.2 (5.9)	0.301
Etiology of CLD					< 0.001
ALD, *n* (%)	240 (36.2%)	13 (11.4%)	52 (30.6%)	175 (46.2%)	
Viral, *n* (%)	238 (35.9%)	64 (56.1%)	76 (44.7%)	98 (25.9%)	
NASH, *n* (%)	43 (6.5%)	6 (5.3%)	12 (7.1%)	25 (6.6%)	
Cryptogenic, *n* (%)	92 (13.9%)	13 (11.4%)	19 (11.2%)	60 (15.8%)	
PBC/PSC, *n* (%)	23 (3.5%)	9 (7.9%)	6 (3.5%)	8 (2.1%)	
AIH, *n* (%)	16 (2.4%)	5 (4.4%)	3 (1.8%)	8 (2.1%)	
Other, *n* (%)	11 (1.6%)	4 (3.5%)	2 (1.2%)	5 (1.3%)	
MELD, median (IQR)	11 (6)	9 (3)	10 (4)	12 (5)	< 0.001
Decompensated ACLD, *n* (%)	356 (53.7%)	19 (16.7%)	67 (39.4%)	270 (71.2%)	< 0.001
Severe/refractory ascites, *n* (%)	132 (19.9%)	3 (2.6%)	15 (8.8%)	76 (20.1%)	< 0.001
History of variceal bleeding, *n* (%)	94 (14.2%)	8 (7.0%)	18 (10.6%)	106 (28.0%)	< 0.001
CTP score, median (IQR)	6 (4)	5 (1)	6 (2)	8 (3)	< 0.001
Child-A, *n* (%)	343 (51.7%)	96 (84.2%)	120 (70.6%)	127 (33.5%)	< 0.001
Child-B, *n* (%)	211 (31.8%)	14 (12.3%)	35 (20.6%)	162 (42.7%)	
Child-C, *n* (%)	109 (16.5%)	4 (3.5%)	15 (8.8%)	90 (23.7%)	
Albumin, g/L (IQR)	36.0 (8.8)	40.1 (5.5)	38.4 (7.0)	33.4 (7.9)	< 0.001
Bilirubin, mg/dL (IQR)	1.2 (1.4)	0.8 (0.7)	0.94 (0.77)	1.47 (1.82)	< 0.001
INR, median (IQR)	1.3 (0.3)	1.2 (0.3)	1.2 (0.3)	1.3 (0.3)	< 0.001
Creatinine, mg/dL (IQR)	0.8 (0.3)	0.8 (0.3)	0.7 (0.3)	0.8 (0.3)	0.218
Sodium, mmol/L (IQR)	138.0 (5.0)	139.0 (3.0)	138.0 (4.0)	138.0 (5.0)	< 0.001
Intake of diuretics, *n* (%)	335 (50.5%)	25 (21.9%)	69 (40.6%)	241 (63.6%)	< 0.001
Intake of ACEi/ARB, *n* (%)	90 (13.6%)	26 (22.8%)	27 (15.9%)	37 (9.8%)	0.001
Arterial hypertension, *n* (%)	235 (35.4%)	45 (39.5%)	64 (37.6%)	126 (33.2%)	0.317
Heart failure, *n* (%)	31 (4.7%)	5 (4.4%)	7 (4.1%)	19 (5.0%)	0.857
Renin [µIU/mL] (IQR)	37.6 (148.1)	21.0 (50.5)	25.1 (70.9)	65.4 (219.6)	< 0.001
Renin > ULN 39.9 µIU/mL, *n* (%)	311 (48.2%)	37 (33.0%)	63 (38.4%)	211 (57.2%)	< 0.001
proBNP [pg/mL] (IQR)^†^	131.7 (294.6)	86.1 (134.0)	63.6 (118.0)	132.2 (208.9)	0.002
proBNP > ULN 125.0 pg/mL, *n* (%)^†^	142 (50.5%)	17 (40.5%)	31 (41.9%)	94 (57.0%)	0.036
Copeptin [pmol/L] (IQR)^‡^	10.3 (21.8)	7.8 (7.7)	5.6 (8.0)	10.7 (18.6)	0.024
Copeptin > ULN 11.4 pmol/L, *n* (%)^‡^	62 (45.6%)	11 (44.0%)	11 (31.4%)	40 (52.6%)	0.112
Median follow-up, months (IQR)	26.2 (40.4)				
Liver transplantation, *n* (%)	51 (7.9%)	4 (3.5%)	8 (4.8%)	39 (10.6%)	0.009
Death, *n* (%)	161 (24.8%)	20 (17.5%)	35 (21.1%)	106 (28.8%)	0.015
Liver-related death, *n* (%)	133 (20.5%)	15 (13.2%)	27 (16.3%)	91 (24.7%)	0.005

^†^proBNP levels are available in 281 patients (HVPG 6–9 mmHg: 42, 10–15 mmHg: 74, ≥ 16 mmHg: 165)

^‡^copeptin levels are available in 136 patients (HVPG 6–9 mmHg: 25, 10–15 mmHg: 35, ≥ 16 mmHg: 76)

A total number of *n* = 663 patients with a median age of 56.6 [IQR 15.5] years and male predominance (*n* = 452; 68.2%) were included. 307 (46.2%) patients had compensated (cACLD) and 356 (53.7%) decompensated (dACLD) cirrhosis. CTP distribution was stage A in 51.7% (*n* = 343), B in 31.8% (*n* = 211), and C in 16.5% (*n* = 109). Median MELD was 11 (IQR 6) points. Stratified by severity of portal hypertension, *n* = 114 (17.2%) patients had HVPG values of 6–9 mmHg, *n* = 170 (25.6%) had HVPG values of 10–15 mmHg, and *n* = 379 (57.2%) had HVPG values ≥ 16 mmHg, respectively. Notably, echocardiography was performed in 219 (33.0%) patients. Only 4 (1.8%) and 2 (0.9%) patients showed moderately to highly reduced left or right ventricular dysfunction, respectively*.* Additional information concerning characteristics of cACLD and dACLD patients, comorbidities and concomitant medication are provided in the supplementary material.

### Progression of portal hypertension is paralleled by alterations in systemic hemodynamics (Fig. S1)

With increasing severity of portal hypertension (i.e. with higher HVPG strata: 6–9 mmHg vs. 10–15 mmHg vs. ≥ 16 mmHg), significantly higher heart rate (median 71.0 [18.0] bpm vs. 76.0 [19.0] bpm vs. 80.0 [22.0] bpm; *p* < 0.001), lower MAP (103.0 [13.5] mmHg vs. 101.0 [19.5] mmHg vs. 99.0 [21.0] mmHg; *p* = 0.032) and lower serum sodium levels (139.0 [3.0] mmol/L vs. 138.0 [4.0] mmol/L vs. 138.0 [5.0] mmol/L; *p* < 0.001) were observed. These trends indicate a progressive state of hyperdynamic circulation with increasing severity of PH. Similar results were obtained for heart rate (74.0 [20.0] bpm vs. 80.0 [21.5] bpm vs. 85.0 [22.0] bpm; *p* < 0.001), MAP (105.0 [18.0] mmHg vs. 98.0 [20.0] mmHg vs. 91.0 [19.0] mmHg; *p* < 0.001) and serum sodium levels (139.0 [3.0] mmol/L vs. 137.0 [5.8] mmol/L vs. 135.0 [7.0] mmol/L, *p* < 0.001) when stratifying by CTP stages (A vs. B vs. C, respectively). HVPG did not correlate with ejection fraction (*n* = 216; *p* = 0.548) or inferior vena cava (IVC) diameter (*n* = 64; *p* = 0.412).

### Association of the severity of portal hypertension with renin, proBNP and copeptin levels (Fig. 1)

**Fig. 1:**
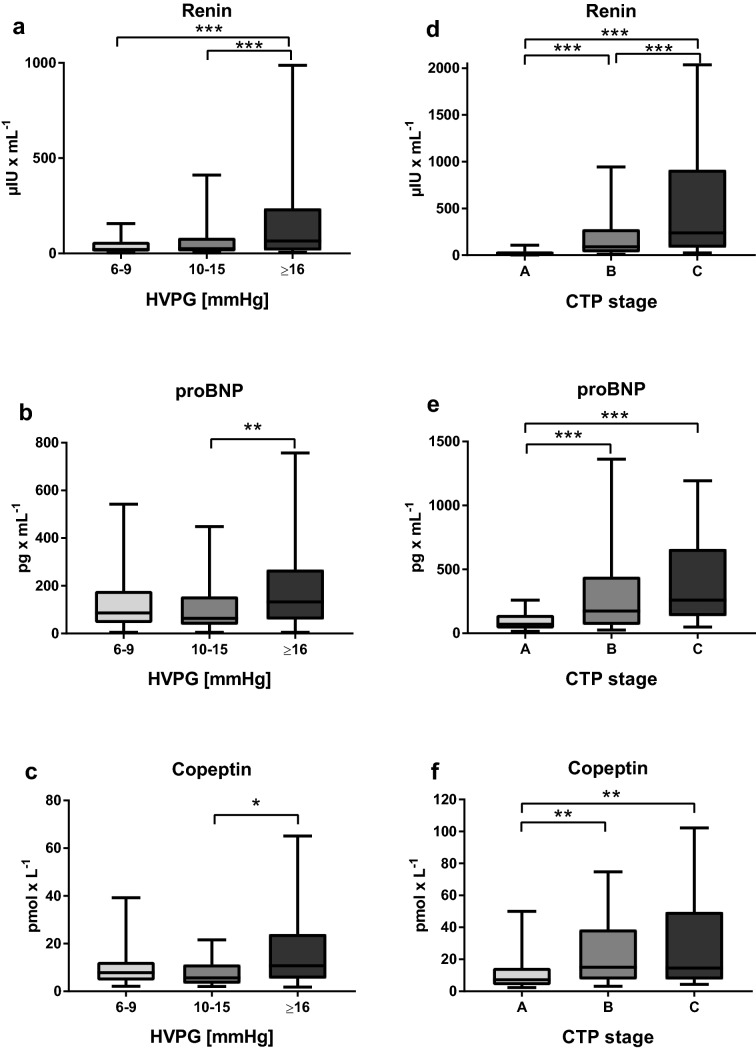
Plasma levels of renin, proBNP and copeptin stratified for HVPG (a–c) and CTP stage (d–f). a, d–f The borders of the whiskers are the 10th and the 90th percentile. b, c Depiction of plasma levels after outlier exclusion, the borders of the whiskers are the minimum and maximum. *HVPG* hepatic venous pressure gradient, *CTP* Child–Turcotte–Pugh, *proBNP* probrain-type natriuretic peptide; *p < 0.050; **p < 0.010; ***p < 0.001

The median renin (*n* = 645) level was 37.6 [148.9] µIU/mL, median proBNP (*n* = 281) level was 131.7 [294.6] pg/mL and median copeptin (*n* = 136) level was 10.3 [21.8] pmol/L. Of note, these levels were mostly within the reported normal ranges for renin at < 39.9 µIU/mL, for proBNP at < 125 pg/mL, and under the determined threshold for copeptin at < 11.4 pmol/L.

Renin levels (6–9 mmHg: 21.0 [50.5] μIU/mL vs. 10–15 mmHg: 25.1 [70.9] μIU/mL vs. ≥ 16 mmHg: 65.4 [219.6] μIU/mL, *p* < 0.001) gradually and significantly increased with HVPG. However, there was no significant difference in renin levels between the HVPG 6–9 mmHg and the HVPG 10–15 mmHg groups.

Median plasma levels of proBNP (6–9 mmHg: 86.1 [134.0] pg/mL vs. 10–15 mmHg: 63.6 [118.0] pg/mL vs. ≥ 16 mmHg: 132.2 [208.9] pg/mL; *p* = 0.002) and copeptin (6–9 mmHg: 7.8 [7.7] pmol/L vs. 10–15 mmHg: 5.6 [8.0] pmol/L vs. ≥ 16 mmHg: 10.7 [18.6] pmol/L, *p* = 0.024) increased with severity of PH—although the findings were not very consistent for both proBNP and copeptin, and significant increases were only observed in the HVPG ≥ 16 mmHg vs. HVPG 10–15 mmHg strata.

### Biomarkers of circulatory homeostasis across Child stages and MELD strata (Fig. 1), in hyponatremia and hypotension (Fig. S2)

Hepatic dysfunction impacted on all three main parameters, as plasma levels of renin (Child-A: 17.7 [31.4] µIU/mL vs. B: 89.8 [245.2] μIU/mL vs. C: 238.0 [833.1] μIU/mL, *p* < 0.001), proBNP (Child-A: 70.3 [105.7] pg/mL vs. B: 174.5 [375.6] pg/mL vs. C: 259.2 [524.6] pg/mL, *p* < 0.001), as well as copeptin (CTP A: 7.3 [10.8] pmol/L vs. B: 15.1 [31.2] pmol/L vs. C: 14.5 [42.1] pmol/L, *p* < 0.001) increased significantly with CTP stage. The same results were obtained when stratifying for MELD: Renin (6–9: 20.6 [45.0] µIU/mL vs. 10–15: 40.3 [176.6] µIU/mL vs. ≥ 16: 147.2 [476.2] µIU/mL; *p* < 0.001), proBNP (6–9: 86.1 [109.2] pg/mL vs. 10–15: 133.2 [273.8] pg/mL vs. 283.9 [700.8] pg/mL; *p* < 0.001) and copeptin levels (6–9: 7.3 [9.5] pmol/L vs. 10–15: 9.4 [16.2] pmol/L vs. ≥ 16: 27.1 [42.6] pmol/L; *p* < 0.001) increased throughout the strata.

In patients with hyponatremia < 130 mmol/L, significantly elevated levels of renin (< 130 mmol/L: 1283.0 [2204.2] μIU/mL vs. ≥ 130 mmol/L: 34.6 [120.0] μIU/mL, *p* < 0.001) and copeptin (< 130 mmol/L: 32.3 [19.2] pmol/L vs. ≥ 130 mmol/L: 9.9 [17.3] pmol/L, *p* = 0.023) were recorded, while there was no significant difference in plasma levels of proBNP (< 130 mmol/L: 156.0 [302.4] pg/mL vs. ≥ 130 mmol/L: 129.0 [289.9] pg/mL, *p* = 0.797) (Fig. S2). Patients with arterial hypotension, as defined by a MAP < 82 mmHg, exhibited increased plasma levels of renin (< 82 mmHg: 124.7 [326.9] μIU/mL vs. ≥ 82 mmHg: 32.4 [121.6] μIU/mL, *p* < 0.001) and proBNP (< 82 mmHg: 151.7 [236.1] pg/mL vs. ≥ 82 mmHg: 94.7 [181.8] pg/mL, *p* = 0.044), whereas there was no significant difference in plasma levels of copeptin (< 82 mmHg: 12.4 [20.1] pmol/L vs. ≥ 82 mmHg: 10.0 [21.9] pmol/L, *p* = 0.748).

### Analysis of independent determinants of renin, proBNP and copeptin levels in ACLD patients (Table 2)

**Table 2:** Assessment of independent determinants of plasma levels of (a) renin, (b) proBNP and (c) copeptin by multiple linear regression analysis ([i] model including MELD and albumin; [ii] model including CTP score and creatinine)

	(a) Renin (*n* = 645)				(b) proBNP (*n* = 281)				(c) Copeptin (*n* = 136)			
	(i)		(ii)		(i)		(ii)		(i)		(ii)	
	aB	*p*	aB	*p*	aB	*p*	aB	*p*	aB	*p*	aB	*p*
Age, 10 years	–	–	–	–	–	–	–	–	–	–	–	–
Sex (male)	–	–	–	–	–	–	–	–	18.9	0.081	13.3	0.208
MELD, points	19.4	0.020	–	–	113.2	< 0.001	–	–	1.8	0.008	–	–
CTP score, points	–	–	10.3	0.571	–	–	137.5	< 0.001	–	–	–	–
HVPG, mmHg	12.8	0.015	13.6	0.005	–	–	–	–	–	–	–	–
Albumin, g/L	10.5	0.088	–	–	12.9	0.421	–	–	− 0.3	0.712	–	–
Creatinine, mg/dL	–	–	279.1	0.054	–	–	794.8	< 0.001	–	–	17.0	< 0.001
Sodium, mmol/L	− 87.2	< 0.001	− 86.5	< 0.001	–	–	–	–	− 1.5	0.118	− 1.9	0.030
Arterial hypertension, yes	–	–	–	–	–	–	–	–	–	–	–	–
Diabetes mellitus, yes	–	–	–	–	–	–	–	–	–	–	–	–
Coronary heart disease, yes	–	–	–	–	–	–	–	–	–	–	–	–
Heart failure, yes	–	–	–	–	–	–	–	–	–	–	–	–

Multivariate analysis showed that plasma levels of renin were independently correlated with hepatic dysfunction (MELD [points; B: 19.4; *p* = 0.020]), HVPG (mmHg; B: 12.8; *p* = 0.015/B: 13.6; *p* = 0.005) and serum sodium (mmol/L; B: − 87.2; *p* < 0.001/B: − 86.5; *p* < 0.001). Plasma levels of proBNP were independently linked to hepatic dysfunction (MELD [points; B: 113.2; *p* < 0.001], CTP score [points; B: 137.5; *p* < 0.001]) and creatinine (mg/dL; B: 794.8; *p* < 0.001). Finally, copeptin plasma levels were independently correlated with liver dysfunction (MELD [points; B: 1.8; *p* = 0.008]), creatinine (mg/dL; B: 17.0; *p* < 0.001) and serum sodium (mmol/L; B: − 1.9; *p* = 0.030). Independent determinants of renin, proBNP and copeptin levels in cACLD and dACLD are depicted in supplementary Tables S5 and S6.

### Impact of renin, proBNP and copeptin on the risk of hepatic decompensation and mortality in cACLD and dACLD (Fig. 2; Table 3)

**Fig. 2:**
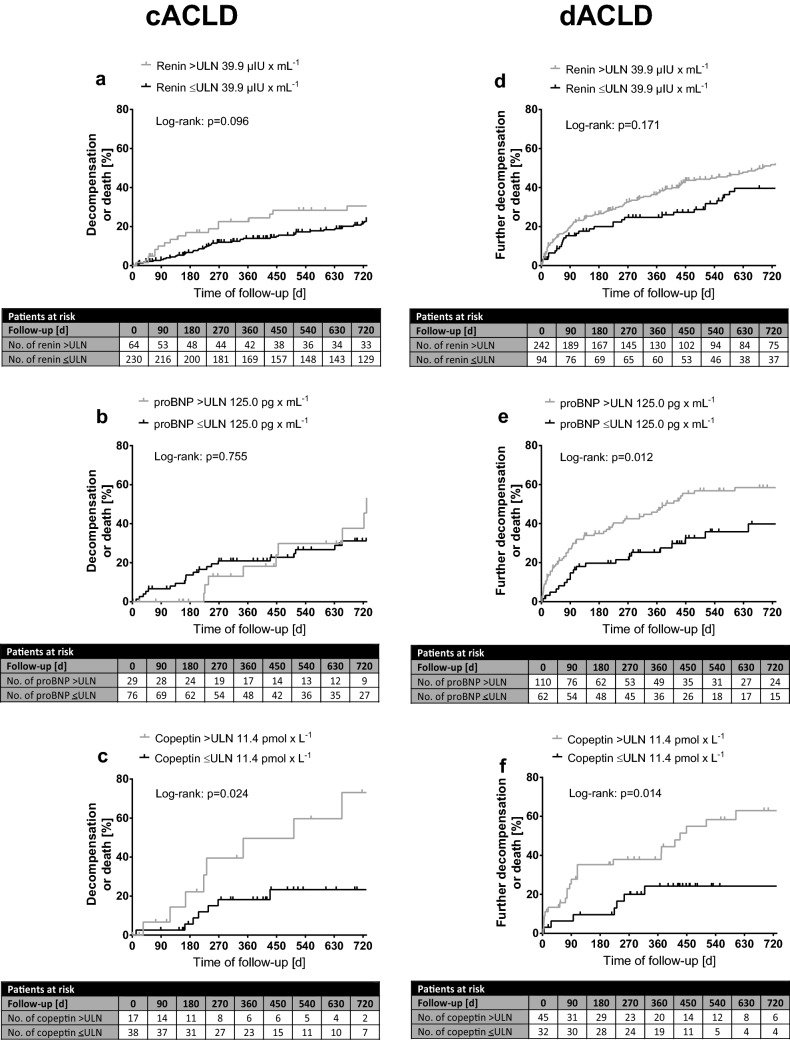
Incidence of first decompensation in **a**–**c** cACLD patients binary for elevated/non-elevated renin, proBNP and copeptin plasma levels. **d**–**f** Incidence of further decompensation in dACLD patients. Time to any first/further decompensation event (development/worsening of ascites or HE, or development of variceal bleeding), death or end of follow-up. Log-rank test is used to determine differences between the groups. *proBNP* probrain-type natriuretic peptide, *ULN* upper limit of normal

**Table 3:** Independent risk factors for first decompensation in cACLD and for further decompensation in dACLD patients

cACLD	(i)			(ii) Renin (*n* = 294)			(iii) proBNP (*n* = 105)			(iv) Copeptin (*n* = 55)		
	HR	95% CI	*p*	aHR	95% CI	*p*	aHR	95% CI	*p*	aHR	95% CI	*p*
Renin, > ULN 39.9 µIU/mL	1.46	0.93–2.28	0.098	1.69	1.07–2.68	0.025	–	–	–	–	–	–
proBNP, > ULN 125.0 pg/mL	0.89	0.44–1.81	0.755	–	–	–	0.75	0.36–1.56	0.433	–	–	–
Copeptin, > ULN 11.4 pmol/L	2.95	1.10–7.90	0.031	–	–	–	–	–	–	2.69	0.99–7.33	0.053
Age, 10 years	1.27	1.07–1.50	0.005	1.31	1.11–1.55	0.002	1.09	0.82–1.45	0.544	1.11	0.77–1.60	0.588
Sex (male)	0.92	0.61–1.37	0.674	–	–	–	–	–	–	–	–	–
MELD, points	1.13	1.08–1.19	< 0.001	1.10	1.03–1.17	0.003	1.16	1.05–1.29	0.004	1.13	0.99–1.29	0.067
HVPG, mmHg	1.07	1.03–1.10	< 0.001	1.06	1.02–1.10	0.005	0.97	0.90–1.05	0.461	0.952	0.86–1.05	0.336
Albumin, g/L	0.91	0.88–0.95	< 0.001	0.95	0.90–1.00	0.017	0.96	0.89–1.03	0.276	0.92	0.81–1.04	0.190
Sodium, mmol/L	0.95	0.90–1.01	0.116	–	–	–	–	–	–	–	–	–

dACLD	(i)			(ii) Renin (*n* = 336)			(iii) proBNP (*n* = 172)			(iv) Copeptin (*n* = 77)		
	HR	95% CI	*p*	aHR	95% CI	*p*	aHR	95% CI	*p*	aHR	95% CI	*p*
Renin, > ULN 39.9 µIU/mL	1.27	0.90–1.77	0.172	1.03	0.73–1.46	0.850	–	–	–	–	–	–
proBNP, > ULN 125.0 pg/mL	1.83	1.13–2.95	0.013	–	–	–	1.34	0.81–2.21	0.260	–	–	–
Copeptin, > ULN 11.4 pmol/L	2.76	1.18–6.44	0.019	–	–	–	–	–	–	2.06	0.84–5.07	0.117
Age, 10 years	1.21	1.06–1.37	0.005	1.24	1.08–1.42	0.003	1.33	1.09–1.62	0.006	1.378	0.99–1.91	0.056
Sex (male)	1.28	0.92–1.77	0.144	–	–	–	–	–	–	–	–	–
MELD, points	1.06	1.03–1.09	< 0.001	1.05	1.02–1.09	0.005	1.08	1.04–1.12	< 0.001	1.14	1.08–1.21	< 0.001
HVPG, mmHg	1.04	1.02–1.06	< 0.001	1.03	1.01–1.05	0.001	1.03	1.00–1.05	0.028	1.01	0.97–1.04	0.780
Albumin, g/L	0.96	0.94–0.98	< 0.001	0.98	0.96–1.00	0.063	0.99	0.96–1.03	0.719	0.98	0.92–1.04	0.449
Sodium, mmol/L	0.98	0.95–1.01	0.212	–	–	–	–	–	–	–	–	–

Next to the univariate analysis (i), multivariate cox regression models for (ii) renin, (iii) proBNP and (iv) copeptin are shown

Follow-up data were available in 648 (cACLD: *n* = 302; dACLD: *n* = 346) patients. Median follow-up time was 26.2 [40.4] months. In total, 225 (34.7%) patients had at least one event of hepatic decompensation during follow-up (70 patients with variceal bleeding, 70 with overt HE and 176 with ascites-related complications; Fig. S3).

Eighty-nine (29.5%) cACLD patients experienced first hepatic decompensation during follow-up (12 variceal bleedings, seven episodes of overt HE and 70 occurrences of ascites). Time to first decompensation was shorter in cACLD patients with elevated renin levels (*n* = 64/294; *p* = 0.096), while there was no difference between patients with elevated (*n* = 29/105) and non-elevated proBNP plasma levels (*p* = 0.755). Importantly, cACLD patients with elevated plasma levels of copeptin (*n* = 17/55; *p* = 0.024) had significantly higher risk of first hepatic decompensation. Cox regression analysis adjusted for age, MELD, HVPG and albumin revealed that elevated plasma level of renin (> 39.9 µIU/mL; hazard ratio [HR]: 1.69; 95% confidence interval [95% CI] 1.07–2.68; *p* = 0.025) independently predicted first hepatic decompensation, while there was a trend for elevated copeptin levels (> 11.4 pmol/L; HR: 2.69; 95% CI 0.99–7.33; *p* = 0.053). In contrast, patients with elevated proBNP plasma levels (> 125 pg/mL; HR: 0.75; 95% CI 0.36–1.56; *p* = 0.433) were not at a higher risk for first hepatic decompensation.

Further decompensation occurred in 134 (38.6%) dACLD patients (34 variceal bleedings, 28 admissions due to overt HE and 72 worsening of ascites events). In dACLD patients, elevated proBNP (*n* = 110/172; *p* = 0.012) and copeptin levels (*n* = 45/77; *p* = 0.014) were associated with significantly shorter time to further decompensation, while there was no difference in time to further decompensation between dACLD patients with elevated (*n* = 242/336) and non-elevated plasma levels of renin (*p* = 0.171). Neither increased renin (HR: 1.03; 95% CI 0.73–1.46; *p* = 0.850) or proBNP (HR: 1.34; 95% CI 0.81–2.21; *p* = 0.260), nor copeptin levels (HR: 2.06; 95% CI 0.84–5.07; *p* = 0.117) predicted further decompensation after adjusting for age, MELD, HVPG and albumin.

### Transplant-free survival according to increased levels of renin, proBNP and copeptin (Fig. 3; Table 4)

**Fig. 3:**
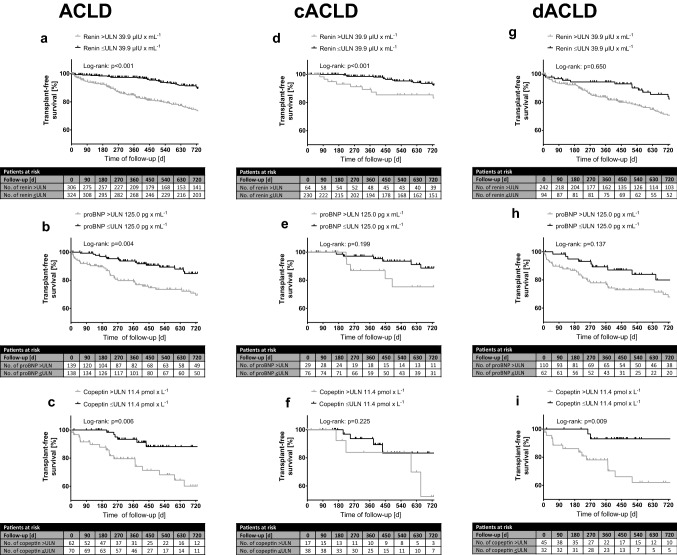
**a-c** Transplant-free mortality in ACLD binary for elevated/non-elevated renin, proBNP and copeptin plasma levels. Transplant-free mortality in **d-f** cACLD patients and in **g-i **dACLD patients. Time to death, liver transplantation or end of follow-up. Log-rank test is used to determine differences between the groups. *proBNP* probrain-type natriuretic peptide, *ULN* upper limit of normal

**Table 4:** Independent risk factors for mortality in the overall cohort, in cACLD and in dACLD patients

Overall cohort	(i)				(ii) Renin (*n* = 630)				(iii) proBNP (*n* = 277)			(iv) Copeptin (*n* = 132)		
	HR	95% CI	*p*		aHR		95% CI	*p*	aHR	95% CI	*p*	aHR	95% CI	*p*
Renin, > ULN 39.9 µIU/mL	2.16	1.57–2.98	< 0.001		1.42		1.01–2.01	0.046	–	–	–	–	–	–
proBNP, > ULN 125.0 pg/mL	2.028	1.24–3.33	0.005		–		–	–	1.30	0.75–2.25	0.349	–	–	–
Copeptin, > ULN 11.4 pmol/L	3.18	1.32–7.67	0.010		–		–	–	–	–	–	3.29	1.36–7.95	0.008
Age, per 10 years	1.43	1.24–1.64	< 0.001		1.40		1.20–1.62	< 0.001	1.46	1.16–1.83	0.001	1.33	0.96–1.84	0.090
Sex (male)	1.39	0.98–1.96	0.065		1.32		0.93–1.90	0.117	1.55	0.91–2.65	0.108	1.79	0.41–7.91	0.441
MELD, points	1.10	1.07–1.13	< 0.001		1.08		1.04–1.12	< 0.001	1.08	1.03–1.13	0.002	1.05	0.98–1.13	0.162
HVPG, mmHg	1.07	1.05–1.09	< 0.001		1.04		1.013–1.057	0.002	1.03	1.00–1.06	0.034	1.00	0.95–1.05	0.955
Albumin, g/L	0.94	0.92–0.96	< 0.001		0.97		0.95–1.00	0.033	1.00	0.96–1.04	0.901	0.97	0.90–1.04	0.363
Sodium, mmol/L	0.94	0.91–0.98	0.002		1.02		0.98–1.07	0.330	1.02	0.97–1.08	0.460	1.03	0.93–1.14	0.566
HCC before baseline	1.561	0.94–2.59	0.084	2.28		1.30–3.40		0.004	0.90	0.30–2.64	0.843	2.36	0.64–8.71	0.198

cACLD	(i)			(ii) Renin (*n* = 294)			(iii) proBNP (*n* = 105)			(iv) Copeptin (*n* = 55)		
	HR	95% CI	*p*	aHR	95% CI	*p*	aHR	95% CI	*p*	aHR	95% CI	*p*
Renin, > ULN 39.9 µIU/mL	2.65	1.47–4.76	0.001	3.15	1.70–5.84	< 0.001	–	–	–	–	–	–
proBNP, > ULN 125.0 pg/mL	1.82	0.72–4.58	0.205	–	–	–	1.02	0.68–1.52	0.943	–	–	–
Copeptin, > ULN 11.4 pmol/L	2.31	0.57–9.33	0.238	–	–	–	–	–	–	2.44	0.59–10.14	0.221
Age, per 10 years	1.54	1.21–1.98	0.001	1.43	1.09–1.86	0.009	1.05	0.71–1.55	0.822	0.96	0.57–1.61	0.868
Sex (male)	0.82	0.45–1.51	0.519	–	–	–	–	–	–	–	–	–
MELD, points	1.12	1.04–1.21	0.002	1.11	1.02–1.21	0.016	0.98	0.81–1.19	0.838	1.03	0.83–1.28	0.782
HVPG, mmHg	1.03	0.98–1.09	0.213	–	–	–	–	–	–	–	–	–
Albumin, g/L	0.94	0.88–0.99	0.029	0.93	0.87–0.99	0.026	0.96	0.86–1.07	0.420	1.05	0.89–1.24	0.563
Sodium, mmol/L	0.99	0.90–1.10	0.875	–	–	–	–	–	–	–	–	–
HCC before baseline	3.00	1.55–5.81	0.001	2.95	1.37–6.34	0.006	1.48	0.48–4.58	0.492	5.32	1.15–24.54	0.032

dACLD	(i)			(ii) Renin (*n* = 336)			(iii) proBNP (*n* = 172)			(iv) Copeptin (*n* = 77)		
	HR	95% CI	*p*	aHR	95% CI	*p*	aHR	95% CI	*p*	aHR	95% CI	*p*
Renin, > ULN 39.9 µIU/mL	1.10	0.73–1.66	0.651	0.89	0.58–1.35	0.567	–	–	–	–	–	–
proBNP, > ULN 125.0 pg/mL	1.61	0.85–3.03	0.141	–	–	–	1.10	0.57–2.15	0.772	–	–	–
Copeptin, > ULN 11.4 pmol/L	5.76	1.30–25.62	0.021	–	–	–	–	–	–	5.77	1.27–26.33	0.024
Age, per 10 years	1.32	1.11–1.58	0.002	1.37	1.14–1.64	0.001	1.59	1.20–2.11	0.001	2.01	1.15–3.51	0.014
Sex (male)	0.71	0.46–1.08	0.105	–	–	–	–	–	–	–	–	–
MELD, points	1.07	1.03–1.10	< 0.001	1.08	1.04–1.12	< 0.001	1.08	1.02–1.14	0.005	1.10	1.01–1.20	0.025
HVPG, mmHg	1.05	1.03–1.08	< 0.001	1.04	1.02–1.07	0.002	1.03	1.00–1.06	0.047	1.00	0.96–1.05	0.949
Albumin, g/L	0.96	0.93–0.98	< 0.001	0.98	0.95–1.01	0.114	1.01	0.97–1.05	0.725	0.95	0.86–1.04	0.259
Sodium, mmol/L	0.98	0.94–1.03	0.457	–	–	–	–	–	–	–	–	–
HCC before baseline	1.24	0.51–3.05	0.633	–	–	–	–	–	–	–	–	–

Next to the univariate analysis (i), multivariate cox regression models for (ii) renin, (iii) proBNP and (iv) copeptin are shown

Fifty-one (7.9%; cACLD: 19 [6.2%]; dACLD: 32 [9.2%]; *p* = 0.189) patients underwent LT and 161 (24.8%; cACLD: 49 [16.2%]; dACLD: 112 [32.4%]; *p* < 0.001) patients died during follow-up with 133 (20.5%; cACLD: 37 [12.2%]; dACLD: 96 [27.7%]; *p* < 0.001) deaths being attributed to liver-related complications (i.e. liver-related mortality).

Elevated levels of renin (*n* = 306/630; *p* < 0.001), proBNP (*n* = 139/277; *p* = 0.004) and copeptin (*n* = 62/132; *p* = 0.006) were associated with shorter transplant-free survival. Univariate analyses showed that increased renin (HR: 2.16; 95% CI 1.57–2.98; *p* < 0.001), proBNP (HR: 2.03; 95% CI 1.24–3.33; *p* = 0.005), and copeptin levels (HR: 3.18; 95% CI 1.32–7.67; *p* = 0.010) predicted mortality. After adjusting for age, sex, MELD, HVPG, albumin, sodium and HCC before baseline, renin (aHR 1.42; 95% CI 1.01–2.01; *p* = 0.046) and copeptin (aHR: 3.29; 95% CI 1.36–7.95; *p* = 0.008) remained as independent predictors of mortality.

Interestingly, within the cACLD cohort, elevated renin plasma levels (*n* = 64/294; *p* < 0.001), but not elevated proBNP (*n* = 29/105; *p* = 0.199) or copeptin levels (*n* = 17/55; *p* = 0.225), were associated with decreased transplant-free survival. Correspondingly, elevated plasma renin independently predicted mortality in cACLD patients adjusted for age, MELD, albumin and HCC before baseline (aHR: 3.15, 95% CI 1.70–5.84; *p* < 0.001), in contrast to elevated proBNP (aHR: 1.02; 95% CI 0.68–1.52; *p* = 0.943) and copeptin levels (aHR: 2.44; 95% CI 0.59–10.14; *p* = 0.221).

In the dACLD cohort, transplant-free survival was not significantly different between patients with normal vs. elevated renin (*n* = 242/346; *p* = 0.650) nor with normal vs. elevated proBNP levels (*n* = 110/175; *p* = 0.137). In contrast, increased copeptin levels (*n* = 45/80; *p* = 0.009) were significantly associated with shorter transplant-free survival. In multivariate Cox regression analysis adjusted for age, MELD, HVPG and albumin, elevated copeptin remained as independent predictor for mortality in dACLD patients (aHR: 5.77; 95% CI 1.27–26.33; *p* = 0.024). There were no independent associations between increased mortality and elevated renin (aHR: 0.89; 95% CI 0.58–1.35; *p* = 0.567) or proBNP levels (aHR: 1.10; 95% CI 0.57–2.15; *p* = 0.772) in the dACLD cohort.

## Discussion

In our thoroughly characterized cohort of ACLD patients, we observed progressive hemodynamic alterations with increasing severity of PH. Stratifying our population not only by hepatic dysfunction (by Child stage and MELD), but also for portal pressure (by the diagnostic gold-standard HVPG), we were able to evaluate the activation of key cardiovascular hormones across the full spectrum of ACLD severity. Renin, proBNP and copeptin levels all increased with severity of PH, indicating activation of all three cardiovascular hormonal systems induced by PH-associated systemic hemodynamic alterations. Notably, the increase of these parameters between patients with HVPG 6–9 mmHg vs. 10–15 mmHg was not significant, suggesting that only pronounced stages of PH have a clinically relevant impact on the RAA, proBNP and AVP pathways. This confirms and goes beyond the findings of Bosch et al. and Arroyo et al. [15, 20], who showed correlations of renin activity and WHVP. It is also in line with previous studies that showed elevated plasma levels of renin, proBNP and copeptin particularly in cirrhotic patients with ascites or other PH-related complications [18, 21, 22]. Considering the implications of RAA activation in the pathogenesis of ascites, these findings explain why HVPG-values ≥ 16 mmHg identify cACLD patients at a particularly high risk for hepatic decompensation [6, 23]. Moreover, renin, proBNP and copeptin levels increased throughout CTP stages and MELD strata, underlining the close correlation of these parameters with the degree of hepatic dysfunction [17, 18, 22]. While there was no correlation of ejection fraction or IVC diameter with severity of portal hypertension, future studies should investigate how changes of the splanchnic/systemic circulation that are primarily caused by portal hypertension, may affect cardiac dysfunction in patients with cirrhosis.

### Renin

Plasma renin levels were significantly increased in patients with arterial hypotension and hyponatremia and renin was independently associated with serum sodium concentration, suggesting renin release upon impaired kidney perfusion or due to reduced sodium concentration sensed by the macula densa [14]. Moreover, renin levels correlated with HVPG, which affirms the association between plasma renin levels and the severity of PH. It has been reported that RAA activation plays a major role in the development of the hemodynamic alterations observed in cirrhotic patients with ascites [21, 24]. However, there is also evidence that RAA is mechanistically involved in splanchnic vasodilation and increased intrahepatic resistance already in early stages of fibrosis and PH [21].

Importantly, cACLD patients with elevated renin plasma levels experienced first hepatic decompensation earlier with plasma renin levels being an independent predictor of first hepatic decompensation, while such an association was not found for further decompensation/death in more advanced dACLD patients. Elevated plasma renin levels were also associated with shorter transplant-free survival and predicted mortality in cACLD, as well as in the overall cohort on adjusted Cox regression analysis. Notably, patients in the cACLD cohort were strictly compensated without (previous) ascites or ascites-related intake of diuretics. These findings are of clinical relevance, as they extend beyond findings of previous studies that reported increased plasma renin levels mostly in patients with ascites [15, 17, 25]. While most (78.2%) of our cACLD cohort had renin levels within the physiological range, our data suggest that a pathological RAA activation may already occur in compensated patients with PH, which is also paralleled by an increased risk for hepatic decompensation and mortality. Since RAA blockade is safe in compensated Child-A patients [26] and also seems to be particularly effective at lowering portal pressure in early ACLD stages [27], the use of RAA biomarkers may identify subgroups of patients, who particularly benefit from RAA blockade.

### Natriuretic peptide proBNP

ProBNP levels increased with advanced liver dysfunction and in pronounced PH (HVPG ≥ 16 mmHg). Increased proBNP levels may indicate a hyperdynamic state in ACLD patients, as they are associated with arterial hypotension and creatinine, potentially linking proBNP to kidney dysfunction. This finding may explain, why elevated proBNP was associated with further decompensation and/or death in already decompensated ACLD patients, although its natriuretic and diuretic functions [13] should actually be beneficial in hyperdynamic ACLD patients by counteracting volume overload. Kidney dysfunction may trigger proBNP secretion through associated volume overload [28], with BNP being insufficient to overcome this volume overload in this particular setting of PH-associated renal dysfunction. Future studies should specifically address the value of proBNP levels in assessing cardiac dysfunction and subsequent renal hypoperfusion and/or acute kidney injury in patients with dACLD. Finally, proBNP levels could also be indicative of cirrhotic cardiomyopathy [29], which is supported by an association of proBNP levels and heart failure in our cohort, as well as a recent study reporting increased risk for post-TIPS heart failure in patients with elevated BNP levels [30].

### Copeptin

Copeptin was negatively associated with serum sodium concentration, indicating an inadequate activation of AVP in ACLD similar to the syndrome of inappropriate antidiuretic hormone secretion (SIADH) [31], causing additional water retention and worsening of dilutional hyponatremia. This hypothesis is in line with our finding of increased copeptin levels in hyponatremic ACLD patients. The activation of AVP in hyperdynamic portal hypertensive patients likely indicates progressive worsening of the hemodynamic state, as previous studies have reported that plasma copeptin may be a surrogate marker for hemodynamic derangement and worse prognosis in patients with ACLD [18, 32]. In our cohort, elevated copeptin levels were linked to shorter time to first hepatic decompensation in cACLD patients, but failed to reach statistical significance on multivariate analysis, potentially due to the small sample size of this subgroup. Moreover, elevated plasma copeptin levels were independently associated with increased mortality in dACLD patients and in the overall ACLD cohort.

### Limitations

A limitation of this study is that proBNP and copeptin were not evaluated in all patients and thus, duration of follow-up was not similar for the different outcome analyses. Additionally, a single assessment of cardiovascular hormones has limitations and is to be seen as additional tool for risk stratification, rather than replacement of HVPG measurement. Moreover, for copeptin, sample size was limited. Intake of diuretics and angiotensin-converting-enzyme inhibitors/angiotensin-1-receptor blockers (ACEi/ARB) was unequally distributed throughout the strata of PH and hepatic dysfunction, as patients with ascites are usually treated with diuretics, while ACEi/ARB are discontinued due to the risk of acute kidney injury. Importantly, proBNP and copeptin levels were not influenced by the intake of diuretics or ACEi/ARB. However, renin levels correlated with diuretic treatment, which was not routinely discontinued before HVPG measurement and blood withdrawal. Of note, no patient was treated with AVP analogues.

## Conclusions

Our results demonstrate a differential activation of renin, natriuretic proBNP and AVP as critical hormones of circulatory homeostasis across distinct stages of PH in patients with ACLD. Increased renin levels predict first decompensation and mortality in ACLD and especially cACLD patients, while elevated copeptin levels were independently associated with mortality, in particular in dACLD patients. Future studies should investigate if these biomarkers may identify ACLD patients who benefit from specific therapeutic interventions that target the molecular signaling axis of these cardiovascular hormones.

## Supplementary Information

Below is the link to the electronic supplementary material.

Supplementary file1 (DOCX 238 kb)

## Data Availability

All authors have access to the entirety of the data underlying this manuscript. Access to the data can be granted at any time upon reasonable request.

## Abbreviations

95% CI: 95% Confidence interval
ACLD: Advanced chronic liver disease
ACEi/ARB: Angiotensin-converting-enzyme inhibitors/angiotensin-1-receptor blockers
aHR: Adjusted hazard ratio
AVP: Arginine-vasopressin
BNP: Brain-type natriuretic peptide
cACLD: Compensated advanced chronic liver disease
CSPH: Clinically significant portal hypertension
CTP: Child–Turcotte–Pugh
dACLD: Decompensated advanced chronic liver disease
EC: Ethics committee
HCC: Hepatocellular carcinoma
HE: Hepatic encephalopathy
HR: Hazard ratio
HVPG: Hepatic venous pressure gradient
IQR: Interquartile range
LT: Liver transplantation
MAP: Mean arterial pressure
MELD: Model for end-stage liver disease
*n*: Number
PH: Portal hypertension
proBNP: Probrain-type natriuretic peptide
RAA: Renin/angiotensin/aldosterone
SIADH: Syndrome of inappropriate antidiuretic hormone secretion
TIPS: Transjugular intrahepatic portosystemic shunt
VIF: Variance inflation factor
WHVP: Wedged hepatic venous pressure
