# Supplementary material for: The differential activation of cardiovascular hormones across distinct stages of portal hypertension predicts clinical outcomes
Source: Hepatol Int. 2021 May 21;15(5):1160–73. doi: 10.1007/s12072-021-10203-9 (PMC8514393; doi:10.1007/s12072-021-10203-9)
Supplement: Supplementary file 1 — Supplementary file1 (DOCX 238 kb) [file 12072_2021_10203_MOESM1_ESM.docx]

**Supplementary information**

**Title: The differential activation of cardiovascular hormones across distinct stages of portal hypertension in patients with cirrhosis predicts clinical outcomes**

Lukas HARTL, MD^1,2^,

Mathias JACHS, MD^1,2^,

Christopher DESBALMES, MD^1,2^

Dunja SCHAUFLER^1,2^

Benedikt SIMBRUNNER, MD^1,2,3^

Rafael PATERNOSTRO, MD^1,2^

Philipp SCHWABL, MD^1,2,3^

David Josef Maria BAUER, MD^1,2^

Georg SEMMLER, MD^1,2^

Bernhard SCHEINER, MD^1,2^

Theresa BUCSICS, MD^1,2^

Ernst EIGENBAUER^4^

Rodrig MARCULESCU, MD^5^

Thomas SZEKERES, MD^5^

Markus PECK-RADOSAVLJEVIC, MD^1,2,6^

Michael TRAUNER, MD^1^

Mattias MANDORFER, MD PhD^1,2^

Thomas REIBERGER, MD^1,2,3^

^1^ Division of Gastroenterology and Hepatology, Department of Medicine III, Medical University of Vienna, Vienna, Austria

^2^Vienna Hepatic Hemodynamic Lab, Medical University of Vienna, Vienna, Austria

^3^Christian Doppler Lab for Portal Hypertension and Liver Fibrosis, Medical University of Vienna, Vienna, Austria

^4^IT-Systems and Communications, Medical University of Vienna, Vienna, Austria

^5^Department of Laboratory Medicine, Medical University of Vienna, Vienna, Austria

^6^Department of Internal Medicine and Gastroenterology (IMuG), Hepatology, Endocrinology, Rheumatology & Nephrology, Central Emergency Medicine (ZAE), Klinikum Klagenfurt am Wörthersee, Klagenfurt, Austria

**Table of contents**

METHODS

Measurement of hepatic venous pressure gradient

Page 5

Laboratory parameters

Page 5

Statistical analysis

Page 6

REFERENCES

Page 7

SUPPLEMENTARY TABLES

-Table-S1. Patient characteristics and comparison between CTP stages

Page 8

-Table-S2. Comparison of HVPG, parameters of circulatory homeostasis, liver function, renal function, medication intake and outcomes between compensated and decompensated patients.

Page 9

-Table-S3. Impact of comorbidities on renin, proBNP and copeptin plasma levels.

Page 10

-Table-S4. Impact of diuretics and ACEi/ARB on renin, proBNP and copeptin plasma levels in all patients, cACLD and dACLD.

Page 11

-Table-S5. Assessment of independent determinants of plasma levels of (a) renin, (b) proBNP and (c) copeptin by multiple linear regression analysis ([i] model including MELD, ascites and albumin; [ii] model including CTP score and creatinine) in cACLD patients.

Page 12

-Table-S6. Assessment of independent determinants of plasma levels of (a) renin, (b) proBNP and (c) copeptin by multiple linear regression analysis ([i] model including MELD, ascites and albumin; [ii] model including CTP score and creatinine) in dACLD patients.

Page 13

SUPPLEMENTARY FIGURES

-Figure-S1. Comparison of (A) MAP, (B) HR, (C) plasma sodium concentration in different stages of PH and in different CTP stages (D-F). The borders of the whiskers are the 10^th^ and the 90^th^ percentile.

Page 14

-Figure-S2. Comparison of (A) renin, (B) proBNP, (C) copeptin levels in patients with/without hyponatremia (sodium <130mmol/L) and in patients with/without severe hypotension (MAP <82mmHg) (D-F). (A-D, F) The borders of the whiskers are the 10^th^ and the 90^th^ percentile. (E) Depiction of plasma levels after outlier exclusion, the borders of the whiskers are the minimum and maximum.

Page 15

-Figure-S3. Depiction of decompensation events during follow-up

Page 16

METHODS

*Measurement of hepatic venous pressure gradient*

For HVPG measurement, a catheter introducer set (8.5 F, Arrow International, Reading, PA, USA) was used to access the right jugular vein via Seldinger technique. Subsequently, a liver vein was cannulated with a balloon catheter (7F, HVPG catheter, Pejcl Medizintechnik, Austria) (1) and HVPG was measured following a standard operating procedure (2) in clinical routine, as supported by the Austrian consensus recommendations (3).

*Laboratory parameters*

Plasma renin concentration was measured by Liaison Direct Renin assay (DiaSorin, Saluggia, Italy), which is a two-sided (sandwich) chemoluminescence assay. For this, magnet particles coated with a highly specific monoclonal antibody, which detects renin and prorenin, as well as a second antibody (specific for renin) conjugated to an isoluminol derivate is used. Renin is bound to the monoclonal antibody connected to the magnet particles, before the isoluminol-antibody conjugate reacts with the bound renin and so-called ‘sandwiches’ – consisting of a renin molecule and both kinds of antibody – are formed. After flushing, the chemoluminescence process is initiated, and the obtained light emitted from the isoluminol-antibody-conjugate is assessed by a photomultiplier in relative light units (RLU), which are directly proportional to the amount of renin in the sample.

For assessment of (NT-)proBNP, the specific immunological test Elecsys proBNP II STAT (Roche, Mannheim, Germany) was used. For this assay, again, two different monoclonal antibodies, one biotinylated and one marked with a ruthenium-complex - are used to form ‘sandwiches’ with proBNP molecules. Streptavidin-coated microparticles are added and the proBNP-sandwiches are bound via biotin-streptavidin-interaction. After this, chemoluminescence emission is induced by applying voltage and measured by photomultiplier. The results are subsequently assessed using a specific master calibration curve.

BRAHMS CT-proAVP LIA (Thermo Scientific Biomarkers, Hennigsdorf, Germany) is the fluorescence-immunoassay that was implemented to measure plasma concentration of copeptin. This assay works by time-resolved amplified cryptate emission (TRACE) technology. For this, a donator (cage-like structure with a terbium-ion in the center) and an acceptor are used. By forming immunocomplexes with copeptin-molecules, donator and acceptor are brought into immediate spatial proximity, leading to a potential energy transfer from donator to acceptor. The sample is then stimulated with a nitrogen laser at 337 nm wavelength. As the donor’s emission spectrum and the acceptor’s absorption spectrum overlap spectrally, the donor’s fluorescence signal is amplified and the acceptor signal’s lifespan is increased, so that a delayed fluorescence measurement becomes possible. This detected delayed signal (in seconds) is directly proportional to the plasma copeptin concentration.

The standard reference values were determined according to the manufacturer’s specifications: Renin: 2.8-39.9 μIU/mL and proBNP: 0-125 pg/mL, while there are no reference values specified for copeptin. Accordingly, the optimal cutoff for transplant-free survival (i.e., 11.4 pmol/L) was determined by Youden’s index. Notably, not all three main parameters were available in all patients. Furthermore, routine laboratory parameters (blood counts, coagulation, chemistry including liver transaminases, creatinine and serum sodium, bilirubin, and albumin) were assessed by standard laboratory methods.”

*Statistical Analysis*

Continuous data was presented as median and interquartile range (IQR), while categorical variables were reported as number (n) of patients and % of these patients with the characteristic of interest. The data sets were tested for normal distribution with D’Agostino & Pearson and Shapiro-Wilk normality test. For comparing continuous variables without normal distribution between two groups, Mann-Whitney U test was used and non-normally distributed continuous variables in three or more groups were compared via Kruskal-Wallis test. Dunn’s multiple comparisons test was implemented as post-hoc test. Group comparisons of categorical variables were conducted using Pearson’s Chi-squared or Fisher’s exact test. Outlier elimination via Rout method (Q=0.1) was conducted when appropriate (4).

Factors associated with renin, proBNP and copeptin plasma levels were assessed using linear regression models. Parameters that showed a trend (p<0.100) were included in the multivariate model. Two separate multivariate models including either (i) MELD and albumin or (ii) CTP and creatinine were implemented. Multicollinearity was investigated via variance inflation factor (VIF).

Kaplan-Meier curves showed the difference in survival between groups of elevated versus non-elevated plasma levels of the parameters of interest. Transplant-free survival time was defined as time to LT, death, or end of follow-up. Patients were censored at the time of LT or end of follow-up. Time to first/further decompensation was defined as time to any first/further decompensation event (development/worsening of ascites or HE, or development of variceal bleeding) or end of follow-up. Differences in survival between these groups were determined by log-rank test. Cox proportional hazard models were computed to assess the impact of renin, proBNP and copeptin levels on first/further decompensation and mortality. Patients entered these models at the time of HVPG measurement. IBM SPSS 22.0 statistic software (IBM, Armonk, NY) and GraphPad Prism 8 (Graphpad Software, La Jolla, CA, USA) were used for statistical analysis. A two-sided p-value of <0.050 was considered as statistically significant.

REFERENCES

1. Ferlitsch A, Bota S, Paternostro R, Reiberger T, Mandorfer M, Heinisch B, Salzl P, et al. Evaluation of a new balloon occlusion catheter specifically designed for measurement of hepatic venous pressure gradient. Liver Int 2015;35:2115-2120.

2. Reiberger T, Schwabl P, Trauner M, Peck-Radosavljevic M, Mandorfer M. Measurement of the Hepatic Venous Pressure Gradient and Transjugular Liver Biopsy. J Vis Exp 2019.

3. Reiberger T, Puspok A, Schoder M, Baumann-Durchschein F, Bucsics T, Datz C, Dolak W, et al. Austrian consensus guidelines on the management and treatment of portal hypertension (Billroth III). Wien Klin Wochenschr 2017;129:135-158.

4. Motulsky H, Brown R. Detecting outliers when fitting data with nonlinear regression – a new method based on robust nonlinear regression and the false discovery rate. BMC Bioinformatics 2006;7.

**Supplementary tables**

**Table-S1. Patient characteristics and comparison between CTP stages**

| Patient characteristics | All patients  (n=663) | CTP A  (n=343) | CTP B  (n=211) | CTP C  (n=109) | p-value |
| --- | --- | --- | --- | --- | --- |
|  |  |  |  |  |  |
| Sex, male/female (% male) | 452/211 (68.2%) | 231/112 (67.3%) | 150/61 (71.1%) | 71/38 (65.1%) | 0.497 |
| Age, years (IQR) | 56.6 (15.5) | 56.0 (14.4) | 58.6 (15.8) | 55.0 (17.9) | **0.049** |
| Etiology of CLD |  |  |  |  | **<0.001** |
| ALD, n (%) | 240 (36.2%) | 62 (18.1%) | 106 (50.2%) | 72 (66.1%) |  |
| Viral, n (%) | 238 (35.9%) | 180 (52.5%) | 43 (20.4%) | 15 (13.8%) |  |
| NASH, n (%) | 43 (6.5%) | 29 (8.5%) | 11 (5.2%) | 3 (1.4%) |  |
| Cryptogenic, n (%) | 92 (13.9%) | 40 (11.7%) | 39 (18.5%) | 13 (6.1%) |  |
| PBC/PSC, n (%) | 23 (3.5%) | 18 (5.2%) | 4 (1.9%) | 1 (0.9%) |  |
| AIH, n (%) | 16 (2.4%) | 7 (2.0%) | 6 (2.8%) | 3 (2.8%) |  |
| Other, n (%) | 11 (1.6%) | 7 (2.0%) | 2 (1.0%) | 2 (1.8%) |  |
| MELD, median (IQR) | 11 (6) | 9 (3) | 12 (5) | 17 (6) | **<0.001** |
| Decompensated ACLD, n (%) | 356 (53,7%) | 70 (20.4%) | 179 (84.8%) | 107 (98.1%) | **<0.001** |
| Severe/refractory ascites, n (%) | 132 (19.9%) | 0 (0%) | 68 (32.2%) | 64 (58.7%) | **<0.001** |
| History of bleeding, n (%) | 94 (14.2%) | 42 (12.2%) | 38 (18.0%) | 14 (12.8%) | 0.145 |
| HVPG, mmHg (IQR) | 17 (10) | 13 (9) | 20 (7) | 21 (9) | **<0.001** |
| 6-9mmHg, n (%) | 114 (17.2%) | 96 (28.0%) | 14 (6.6%) | 4 (3.7%) | **<0.001** |
| 10-15mmHg, n (%) | 170 (25.6%) | 120 (35.0%) | 35 (16.6%) | 15 (13.8%) |  |
| ≥16mmHg, n (%) | 379 (57.2%) | 127 (37.0%) | 162 (76.8%) | 90 (82.6%) |  |
| Albumin, g x L^-1^ (IQR) | 36.0 (8.8) | 39.4 (5.5) | 32.7 (6.2) | 28.3 (7.2) | **<0.001** |
| Bilirubin, mg x dL^-1^ (IQR) | 1.2 (1.4) | 0.9 (0.7) | 1.4 (1.4) | 3.7 (3.9) | **<0.001** |
| INR, median (IQR) | 1.3 (0.3) | 1.2 (0.2) | 1.3 (0.3) | 1.6 (0.5) | **<0.001** |
| Creatinine, mg x dL^-1^ (IQR) | 0.8 (0.3) | 0.7 (0.3) | 0.8 (0.3) | 0.8 (0.4) | **0.001** |
| Sodium, mmol x L^-1^ (IQR) | 138.0 (5.0) | 139 (3) | 137.0 (5.8) | 135 (7) | **<0.001** |
|  |  |  |  |  |  |
| Renin [µIU x mL^-1^] (IQR) | 37.6 (148.1) | 17.7 (31.4) | 89.8 (245.2) | 238.0 (832.9) | **<0.001** |
| Renin >ULN 39.9 µIU x mL^-1^, n (%) | 311 (48.2%) | 82 (24.5%) | 140 (68.0%) | 89 (85.6%) | **<0.001** |
| proBNP [pg x mL^-1^] (IQR)^†^ | 131.7 (294.6) | 70.3 (105.7) | 174.5 (375.6) | 259.2 (524.6) | **<0.001** |
| proBNP >ULN 125.0 pg x mL^-1^, n (%)^†^ | 142 (50.5%) | 32 (27.6%) | 60 (59.4%) | 50 (78.1%) | **<0.001** |
| Copeptin [pmol x L^-1^] (IQR)^‡^ | 10.3 (21.8) | 7.3 (10.8) | 15.1 (31.2) | 14.5 (42.2) | **<0.001** |
| Copeptin > 11.4 pmol x L^-1^, n (%)^‡^ | 62 (45.6%) | 18 (28.1%) | 28 (58.3%) | 16 (66.7%) | **<0.001** |

Information available in:

^†^ proBNP levels are available in 281 patients (CTP-A: 116, CTP-B: 101, CTP-C: 64);

^‡^ copeptin levels are available in 136 patients (CTP-A: 64, CTP-B: 48, CTP-C: 24);

**Table-S2. Comparison of HVPG, parameters of circulatory homeostasis, liver function, renal function and medication intake between compensated and decompensated patients.**

| **Patient characteristics** | **cACLD (n=307)** | **dACLD (n=356)** | **p-value** |
| --- | --- | --- | --- |
|  |  |  |  |
| **MELD, median (IQR)** | 9 (4) | 13 (6) | **<0.001** |
| **HVPG, mmHg (IQR)** | 12 (8) | 20 (8) | **<0.001** |
|  |  |  |  |
| **Renin, µIU x mL^-1^ (IQR)** | 17.1 (28.9) | 112.9 (317.7) | **<0.001** |
| **Renin >ULN 39.9 µIU x mL^-1^, n (%)** | 65 (21.7%) | 246 (71.0%) | **<0.001** |
| **proBNP, pg x mL^-1^ (IQR)****^†^** | 67.9 (104.5) | 228.8 (431.9) | **<0.001** |
| **proBNP >ULN 125.0 pg x mL^-1^, n (%)^†^** | 29 (27.4%) | 113 (64.5%) | **<0.001** |
| **Copeptin, pmol x L^-1^, (IQR)** ^‡^ | 7.5 (11.8) | 13.4 (29.5) | **0.001** |
| **Copeptin > 11.4 pmol x L^-1^, n (%)**^‡^ | 15 (26.8%) | 38 (47.5%) | **0.020** |
|  |  |  |  |
| **Albumin, g x L^-1^ (IQR)** | 38.9 (6.5) | 32.8 (8.5) | **<0.001** |
| **Bilirubin, mg x dL^-1^ (IQR)** | 0.9 (0.8) | 1.5 (2.1) | **<0.001** |
| **INR, median (IQR)** | 1.2 (0.2) | 1.4 (0.4) | **<0.001** |
| **Creatinine, mg x dL^-1^ (IQR)** | 0.7 (0.3) | 0.8 (0.4) | **<0.001** |
| **Sodium, mmol x L^-1^ (IQR)** | 139.0 (3.0) | 137.0 (5.0) | **<0.001** |
| **Diuretics intake, n (%)** | 32 (10.4%) | 303 (85.1%) | **<0.001** |
| **ACEi/ARB intake, n (%)** | 61 (19.9%) | 29 (8.1%) | **<0.001** |

Information available in:

**^†^** proBNP levels are available in 281 patients (cACLD: 106, dACLD: 175);

^‡^ copeptin levels are available in 136 patients (cACLD: 56, dACLD: 80);

**Table-S3. Impact of comorbidities on renin, proBNP and copeptin plasma levels.**

|  | **Arterial hypertension**  **n=236** | **No arterial hypertension**  **n=420** | **p** | **Diabetes mellitus**  **n=158** | **No diabetes mellitus**  **n=497** | **p** | **Coronary heart disease**  **n=59** | **No coronary heart disease**  **n=597** | **p** | **Heart failure**  **n=32** | **No heart failure**  **n=625** | **p** |
| --- | --- | --- | --- | --- | --- | --- | --- | --- | --- | --- | --- | --- |
| **Renin, µIU x mL^-1^ [IQR]** | 33.2 [143.7] | 39.7 [149.0] | 0.279 | 33.3 [135.0] | 38.3 [151.6] | 0.511 | 28.9 [395.4] | 38.1 [134.0] | 0.963 | 46.9 [164.9] | 37.1 [149.5] | 0.869 |
| **proBNP, pg x mL^-1^ [IQR]** | 149.6 [487.2] | 129.0 [255.2] | 0.152 | 124.2 [198.2] | 134.6 [332.2] | 0.424 | 233.5 [1191.6] | 122.3 [257.1] | **0.018** | 231.3 [874.1] | 108.7 [277.9] | **0.005** |
| **Copeptin, pmol x L^-1^ [IQR]** | 12.9 [27.1] | 8.8 [16.1] | 0.053 | 9.1 [11.3] | 11.3 [23.8] | 0.440 | 9.7 [35.6] | 10.8 [20.8] | 0.640 | 40.7 [77.2] | 9.9 [16.4] | **0.008** |

**Table-S4. Impact of diuretics and ACEi/ARB on renin, proBNP and copeptin plasma levels in all patients, cACLD and dACLD.**

| **All patients** | **Diuretics**  **n=335** | **No diuretics**  **n=328** | **p** | **ACEi/ARB**  **n=90** | **No ACEi/ARB**  **n=573** | **p** |
| --- | --- | --- | --- | --- | --- | --- |
| **Renin, µIU x mL^-1^ [IQR]** | 129.7 [353.6] | 17.8 [25.6] | **<0.001** | 43.7 [230.1] | 36.5 [136.1] | 0.539 |
| **proBNP, pg x mL^-1^ [IQR]** | 207.4 [405.3] | 71.3 [112.6] | **<0.001** | 104.8 [664.7] | 132.7 [275.7] | 0.769 |
| **Copeptin, pmol x L^-1^ [IQR]** | 15.6 [28.4] | 7.5 [9.7] | **<0.001** | 7.6 [12.2] | 10.8 [22.2] | 0.596 |
| **cACLD** | **Diuretics**  **n=32** | **No diuretics**  **n=275** | **p** | **ACEi/ARB**  **n=61** | **No ACEi/ARB**  **n=246** | **p** |
| **Renin, µIU x mL^-1^ [IQR]** | 37.6 [121.6] | 16.45 [24.2] | **<0.001** | 25.1 [105.6] | 15.6 [21.3] | **0.001** |
| **proBNP, pg x mL^-1^ [IQR]** | 67.4 [140.2] | 67.9 [99.4] | 0.717 | 79.6 [183.4] | 66.3 [93.1] | 0.193 |
| **Copeptin, pmol x L^-1^ [IQR]** | 13.6 [11.4] | 7.0 [11.1] | 0.377 | 7.6 [12.0] | 7.3 [12.1] | 0.538 |
| **dACLD** | **Diuretics**  **n=303** | **No diuretics**  **n=53** | **p** | **ACEi/ARB**  **n=29** | **No ACEi/ARB**  **n=327** | **p** |
| **Renin, µIU x mL^-1^ [IQR]** | 145.6 [357.6] | 23.8 [37.9] | **<0.001** | 250.5 [1304.5] | 108.9 [268.9] | 0.176 |
| **proBNP, pg x mL^-1^ [IQR]** | 229.8 [421.5] | 107.9 [516.0] | 0.334 | 535.2 [1295.9] | 227.0 [396.0] | 0.216 |
| **Copeptin, pmol x L^-1^ [IQR]** | 15.6 [30.2] | 8.6 [17.4] | 0.160 | 6.7 [58.5] | 13.4 [28.5] | 0.766 |

**Table-S5. Assessment of independent determinants of plasma levels of (a) renin, (b) proBNP and (c) copeptin by multiple linear regression analysis ([i] model including MELD, ascites and albumin; [ii] model including CTP score and creatinine) in cACLD patients.**

| **cACLD** | **(a) Renin (n=299)** | | | | **(b) proBNP (n=106)** | | | | **(c) Copeptin (n=56)** | | | |
| --- | --- | --- | --- | --- | --- | --- | --- | --- | --- | --- | --- | --- |
|  | **(i)** | | **(ii)** | | **(i)** | | **(ii)** | | **(i)** | | **(ii)** | |
|  | **aB** | **p** | **aB** | **p** | **aB** | **p** | **aB** | **p** | **aB** | **p** | **aB** | **p** |
| **Age, per 10 years** | - | - | - | - | 114.0 | **0.003** | 46.2 | 0.095 | - | - | - | - |
| **Sex (male)** | 31.7 | 0.121 | 34.4 | 0.094 | - | - | - | - | - | - | - | - |
| **MELD, points** | 8.3 | **0.008** | - | - | 43.0 | **0.010** | - | - | - | - | - | - |
| **CTP score, points** | - | - | - | - | - | - | - | - | - | - | 19.5 | **0.029** |
| **HVPG, mmHg** | - | - | - | - | - | - | - | - | - | - | - | - |
| **Albumin, g x L^-1^** | - | - | - | - | - | **-** | - | - | -2.9 | 0.079 | - | - |
| **Creatinine, mg x dL^-1^** | - | - | - | - | - | - | 564.5 | **<0.001** | - | - | - | - |
| **Sodium, mmol x L^-1^** | -5.2 | 0.147 | -6.4 | 0.077 | - | - | - | - | -6.8 | **0.007** | -6.0 | **0.016** |
| **Arterial hypertension, yes** | - | **-** | - | **-** | - | **-** | - | **-** | - | - | - | - |
| **Diabetes mellitus, yes** | - | **-** | - | **-** | - | **-** | - | **-** | - | - | - | - |
| **Coronary heart disease, yes** | - | **-** | - | **-** | - | - | - | - | - | - | - | - |
| **Heart failure, yes** | - | **-** | - | **-** | - | **-** | - | **-** | - | - | - | - |

**Table-S6. Assessment of independent determinants of plasma levels of (a) renin, (b) proBNP and (c) copeptin by multiple linear regression analysis ([i] model including MELD, ascites and albumin; [ii] model including CTP score and creatinine) in dACLD patients.**

| **dACLD** | **(a) Renin (n=346)** | | | | **(b) proBNP (n=175)** | | | | **(c) Copeptin (n=80)** | | | |
| --- | --- | --- | --- | --- | --- | --- | --- | --- | --- | --- | --- | --- |
|  | **(i)** | | **(ii)** | | **(i)** | | **(ii)** | | **(i)** | | **(ii)** | |
|  | **aB** | **p** | **aB** | **p** | **aB** | **p** | **aB** | **p** | **aB** | **p** | **aB** | **p** |
| **Age, per 10 years** | - | - | **-** | **-** | - | - | - | - | - | - | - | - |
| **Sex (male)** | - | - | **-** | **-** | - | - | - | - | 19.6 | 0.068 | 12.4 | 0.222 |
| **MELD, points** | 14.8 | 0.228 | **-** | **-** | 135.8 | **<0.001** | - | - | 1.8 | **0.004** | - | - |
| **CTP score, points** | - | - | -12.7 | 0.680 | - | - | 199.7 | **0.003** | - | - | - | - |
| **HVPG, mmHg** | 17.1 | 0.052 | 17.1 | 0.052 | - | - | - | - | - | - | - | - |
| **Albumin, g x L^-1^** | - | - | **-** | **-** | - | - | - | - | - | - | - | - |
| **Creatinine, mg x dL^-1^** | - | - | **-** | **-** | - | - | 824.9 | **<0.001** | - | - | 19.8 | **<0.001** |
| **Sodium, mmol x L^-1^** | -109.6 | **<0.001** | -109.6 | **<0.001** | 63.4 | **0.029** | 60.5 | **0.043** | - | - | - | - |
| **Arterial hypertension, yes** | - | **-** | - | **-** | - | **-** | - | **-** | - | - | - | - |
| **Diabetes mellitus, yes** | - | **-** | - | **-** | - | **-** | - | **-** | - | - | - | - |
| **Coronary heart disease, yes** | - | **-** | - | **-** | 1279.0 | **0.003** | 968.4 | **0.026** | - | - | - | - |
| **Heart failure, yes** | - | **-** | - | **-** | - | **-** | - | **-** | - | - | - | - |

**Supplementary Figures**

**Figure-S1. Comparison of (A) MAP, (B) heart rate, (C) plasma sodium concentration in different stages of PH and (D-F) in different CTP stages.** The borders of the whiskers are the 10^th^ and the 90^th^ percentile

*Abbreviations: MAP=mean arterial pressure; HVPG=hepatic venous pressure gradient; CTP=Child-Turcotte-Pugh, proBNP=probrain-type natriuretic peptide; * p<0.050; ** p<0.010; *** p<0.001*

**Figure-S2. Comparison of (A) renin, (B) proBNP, (C) copeptin levels in patients with/without hyponatremia (sodium <130mmol/L) and (D-F) in patients with/without severe hypotension (MAP <82mmHg).** (A-D, F) The borders of the whiskers are the 10^th^ and the 90^th^ percentile. (E) Depiction of plasma levels after outlier exclusion, the borders of the whiskers are the minimum and maximum

*Abbreviations: MAP=mean arterial pressure; proBNP=probrain-type natriuretic peptide; * p<0.050; ** p<0.010; *** p<0.001*

**Figure-S3. Depiction of decompensation events during follow-up.**

*Abbreviations: AS=ascites; HE=hepatic encephalopathy; VB=variceal bleeding*

**
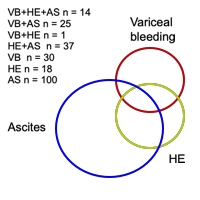
**
